# Humoral and cellular immunity against different SARS-CoV-2 variants in patients with chronic kidney disease

**DOI:** 10.1038/s41598-023-47130-8

**Published:** 2023-11-15

**Authors:** Desmond Yat-Hin Yap, Carol Ho-Yan Fong, Xiaojuan Zhang, Jonathan Daniel Ip, Wan-Mui Chan, Allen Wing-Ho Chu, Lin-Lei Chen, Yan Zhao, Brian Pui-Chun Chan, Kristine Shik Luk, Vincent Chi-Chung Cheng, Tak-Mao Chan, Kelvin Kai-Wang To

**Affiliations:** 1 Division of Nephrology, Department of Medicine, Queen Mary Hospital, School of Clinical Medicine, Li Ka Shing Faculty of Medicine, The University of Hong Kong, Pokfulam, Hong Kong Special Administrative Region People’s Republic of China; 2 State Key Laboratory for Emerging Infectious Diseases, Carol Yu Centre for Infection, Department of Microbiology, School of Clinical Medicine, Li Ka Shing Faculty of Medicine, The University of Hong Kong, Pokfulam, Hong Kong Special Administrative Region People’s Republic of China; 3 Centre for Virology, Vaccinology and Therapeutics, Hong Kong Science and Technology Park, Shatin, Hong Kong Special Administrative Region People’s Republic of China; 4 Department of Pathology, Princess Margaret Hospital, Kwai Chung, Hong Kong Special Administrative Region People’s Republic of China; 5 Present Address: Department of Microbiology, Queen Mary Hospital, Pokfulam, Hong Kong Special Administrative Region People’s Republic of China; 6 Department of Clinical Microbiology and Infection Control, The University of Hong Kong-Shenzhen Hospital, Shenzhen, People’s Republic of China

**Keywords:** Microbiology, Medical research, Nephrology

## Abstract

Chronic kidney disease (CKD) patients are at higher risk of severe COVID-19. Humoral and cellular immunity from prior infection or vaccination are important for protection, but the neutralizing antibody (nAb) response against SARS-CoV-2 variants is impaired. We investigated the variant-specific nAb and T cell immunity among CKD patients. Adult CKD patients were recruited between August and October 2022. nAb against the SARS-CoV-2 (ancestral strains and four Omicron sublineages) and T cell response were measured using the live virus neutralization assay and interferon-gamma release assay (IGRA). The correlation between nAb/T-cell response and subsequent infection after recruitment were also determined. Among the 88 recruited patients, 95.5% had prior infection or had completed the primary vaccine series. However, only 77.3% had detectable nAb against at least one SARS-CoV-2 strains, 59.1% tested positive in IGRA, and 52.3% had detectable nAb and tested positive in the IGRA. The nAb geometic mean titers (GMTs) against XBB.1, BA.5 and BA.2.3.20 were significantly lower than those against BA.2 and ancestral strain. Prior SARS-CoV-2 infection was associated with elevated nAb and T cell response. More kidney transplant recipients (KTRs) showed absent nAb and T cell response (36.8% vs. 10.1%), despite a higher prevalence of vaccine booster in this population (94.7% vs. 50.7%). Lower levels of nAb titer and T cell response were significantly associated with subsequent infection. A considerable proportion of CKD patients, especially KTRs, showed absence of humoral and cellular protective immunity against SARS-CoV-2. Strategies to improve immunogenicity in this population are urgently needed.

## Introduction

The Coronavirus disease 2019 (COVID-19) pandemic, caused by severe acute respiratory syndrome coronavirus 2 (SARS-CoV-2), conferred significant healthcare and socioeconomic impact worldwide. Patients with chronic kidney disease (CKD) are particularly vulnerable to SARS-CoV-2 infection, with substantially increased rates of severe disease, intensive care unit (ICU) admissions and death when compared to the general population^1^. The adjusted risk ratio of severe disease has been estimated to be 3.71 for patients with stage 5 CKD when compared with patients without CKD^2^. Among CKD patients, those who require long-term renal replacement therapy (RRT), including dialysis patients or kidney transplant recipients (KTRs), are especially susceptible to SARS-CoV-2 infection. Indeed, in a systematic review conducted in 2020, KTRs showed significantly higher overall mortality rates than general patients who require hospitalization (20–40% vs. ~ 10–15%)^3^. Recent studies have reported a mortality rate of 0.8–1.1% in KTRs with SARS-CoV-2 infection after the emergence of the Omicron variant^4–6^.

SARS-CoV-2 infection or COVID-19 vaccination can induce protective humoral and cellular immune response against SARS-CoV-2. The levels of humoral and cellular immunity correlate with protection from reinfection or vaccine breakthrough infections^7–10^, and are dependent on several factors, such as the number of doses of vaccination, the type of vaccine, the time interval between the last dose of vaccine or last episode of infection, and whether the patient had hybrid immunity^11^. The levels of SARS-CoV-2 specific antibody also correlates with protection among patients on dialysis^12^. However, kidney dysfunction is associated with several immune defects which affect the immune response after infection or vaccination, including compromised B cell maturation, enhanced B cell apoptosis, aberrant T cell function, downregulation of toll-like receptors/co-stimulation molecules in immune cell types and the use of immunosuppressive agents in patients with glomerulonephritis (GN) or kidney transplantation^13^. Furthermore, CKD is often associated with immunocompromised conditions, such as systemic lupus erythematosus and diabetes mellitus. Our previous meta-analysis found that the seropositive rate of anti-SARS-CoV-2 antibody in CKD patients after 2 doses of COVID-19 vaccine was 44% lower than the general population^14^. Such reduced immunogenicity to COVID-19 vaccines corresponds to remarkably higher rates of breakthrough infections of dialysis and transplant patients (2.2–17.8%) than that in healthy individuals (4.3%)^15–17^. One recent study also showed that non-dialysis CKD patients had higher risk of breakthrough infections compared to the general population, though not as significant as those on dialysis or transplant^18^. Moreover, previous studies also found that KTRs or patients on hemodialysis (HD) had poorer T cell response after COVID-19 vaccine than the control group^19,20^. The lack of CD8 T cell response correlated with breakthrough infection among KTRs who have received 3 doses of COVID-19 vaccines^21^. Pedersen et al.^22^ reported a renal transplant recipient who did not have detectable levels of neutralizing antibody (nAb) and T cell response after immunization with an mRNA vaccine.

Most of the previous COVID-19 immunity studies among CKD patients were conducted before the Omicron variant appeared. When first appeared in late 2021, the Omicron variant BA.1 and BA.2 sublineages were found to be much more resistant to nAb induced from prior infection or vaccination^23–25^. Throughout 2022 and 2023, the Omicron variant has evolved into several sublineages with increasing immune escape, especially the BA.5 and XBB^11,26^. In this study, we analyzed the antibody and T cell response among CKD patients, and determined the correlation of protection when the Omicron BA.5 and BA.2.3.20 dominated in Hong Kong in late 2022. Unlike previous studies, we determined the nAb response against COVID-19 variants that are circulating at the time of infection since the protection from past infection varies for different variants^27^. Furthermore, we compared patients with or without prior infection because infection with Omicron variants can induce a distinct immune response from patients who have been vaccinated^11^. Our results therefore will provide useful information regarding how well CKD patients are protected against COVID-19 reinfection and help formulate the optimal preventive strategy for these highly susceptible individuals.

## Methods

### Study participants

This is a prospective cross-sectional study of patients with CKD. We recruited patients from the out-patient clinic or dialysis centre of Queen Mary Hospital between August 11 and October 5, 2022. Patients were eligible for inclusion if they were aged 18 years or above, and with one of the following kidney conditions: (1) CKD of stage 3 or above (defined as an estimated glomerular filtration rate [eGFR] of < 60 ml/min for > 3 months); (2) received peritoneal dialysis (PD) for more than 1 month; (3) received HD for more than 1 month; (4) KTRs who were receiving maintenance immunosuppression; (5) immune-mediated GN who were receiving maintenance immunosuppression. Patients were excluded if they had acute kidney injury, refused to provide written informed consent, mental incapacity to provide written informed consent, or unable to contribute sufficient volume of blood. In our centre, we adopted protocolized immunosuppressive regimens for KTRs and patients with immune-mediated GN. KTRs received triple maintenance immunosuppression comprising corticosteroids, calcineurin inhibitor (CNI) and anti-metabolite (mycophenolate mofetil as first-line, and substituted with mTOR inhibitors in patients who developed post-transplant malignancy). Maintenance immunosuppression for patients with immune-mediated GN were given according to the renal pathologies (corticosteroids and anti-metabolite for patients with lupus nephritis, ANCA vasculitis and membranoproliferative GN; corticosteroids with or without CNI for patients with minimal change nephropathy and membranous nephropathy). Blood specimens were collected using clotted blood tube and lithium heparin blood tube. This study was approved by the Institutional Review Board of the University of Hong Kong/Hospital Authority of Hong Kong West Cluster (HKU/HA HKW IRB) (Reference number UW 22–555 and UW 21–313). The research was conducted in accordance with the 1964 Declaration of Helsinki. Written informed consent was obtained from all study participants.

### Definitions

Based on the definition from the World Health Organization, a patient was considered to have completed the primary series of COVID-19 vaccine if they had received at least 2 doses of BNT162b2 or CoronaVac^28^. Patients were classified as being previously infected with SARS-CoV-2 if they had known positive reverse transcription-polymerase chain reaction (RT-PCR) or rapid antigen test results; or tested positive for IgG against SARS-CoV-2 nucleocapsid (N) protein in this study if they had not received inactivated whole virion COVID-19 vaccine previously.

### Viral culture and live virus neutralizing antibody assay

Viral culture was performed with TMPRSS2-expressing VeroE6 (VeroE6/TMPRSS2) cells in a biosafety level 3 facility as we described previously with slight modifications^11,26,29^. Briefly, TMPRSS2-expressing VeroE6 (VeroE6/TMPRSS2) cells (JCRB Cat#JCRB1819) were seeded with 1 mL of minimum essential medium (MEM) (Gibco®, Thermo Fisher Scientific) with 10% fetal bovine serum (FBS) and 1 mg/mL G418 (Gibco®, Thermo Fisher Scientific) at 1 × 10^5^ cells in a shell vial (Diagnostic Hybrids, Inc). The shell vials were incubated at 37 °C with 5% CO_2_ until confluence for inoculation. Each vial was inoculated with 100 μL of clinical specimen. One hour after incubation, the clinical specimen was removed and cells were replenished with 1 mL of MEM medium with 1% FBS, 100 IU/ml penicillin, 100 μg/mL streptomycin, 20 U/ml of nystatin, and 25 mM HEPES (Gibco®, ThermoFisher Scientific). The cells were incubated at 37 °C with 5% CO_2_ and observed daily for virus-induced cytopathic effect (CPE). Cultures with more than 50% CPE were expanded to large volume in VeroE6/TMPRSS2 cells with the same culture condition. The 50% tissue culture infective doses (TCID_50_) were determined in VeroE6/TMPRSS2 cells. The SARS-CoV-2 lineage of the virus culture isolates was confirmed using whole genome sequencing.

Live virus nAb assay was performed as we described previously^11,26,29^. The SARS-CoV-2 ancestral strain (GISAID accession number EPI_ISL_17668119) and four Omicron variant sublineages (BA.2.2 [GISAID accession number EPI_ISL_17668120], BA.2.3.20 [GISAID accession number EPI_ISL_16342299], BA.5.2 [GISAID accession number EPI_ISL_13777658], XBB.1 [GISAID accession number EPI_ISL_15602393]) were included. Briefly, serum samples were heat-inactivated at 56 °C for 30 min and were serially diluted in 2-folds with MEM containing 1% FBS. Duplicates of each diluted serum were mixed with a SARS-CoV-2 virus isolate to reach a final concentration of 100 TCID_50_ and were incubated at 37 °C for 1 h. After incubation, 100 μL of the serum-virus mixture was then added to VeroE6/TMPRSS2 cells that were seeded in 96-well plates 48 h before infection. The cells were incubated with the mixture at 37 °C. After incubation for 3 days, CPE was visually scored for each well by two independent observers. The 50% neutralization titer (NT_50_) was determined by using log (inhibitor) versus normalized response- variable slope in GraphPad PRISM version 9.4.0. For statistical analysis, a value of 5 was assigned if the live virus nAb titer is < 10.

### Interferon-gamma release assay

SARS-CoV-2-specific T cell response was measured using a SARS-CoV-2 spike protein specific quantitative IFN-gamma release assay (IGRA) with whole blood following the manufacturer´s instructions (Wantai SARS-CoV-2 IGRA, Wantai Biopharm, Beijing, China). Lithium heparinized blood collected by VACUETTE® LH lithium heparin tubes (Greiner Bio-One, Kremsmünster, Austria) from each patient was processed on the same day of collection and was incubated 24 h at 37 °C in three tubes supplied: i) Background control culture tube (N tube) for the individual IFN-γ background; (ii) positive control culture tube (P tube) with SARS-CoV-2 non-specific antigen for unspecific IFN-gamma secretion as controls; and (iii) testing culture tube (T tube) with antigens of the SARS-CoV-2 spike protein for specific IFN-gamma secretion. The IFN-gamma concentration released in the plasma fraction obtained after centrifugation of the three tubes was then measured using an enzyme-linked immunosorbent assay (ELISA) provided in the same assay kit. IFN-gamma response was measured with wavelength 620–450 nm by SkanIt Microplate Readers and analyzed with SkanIt Software for Microplate Readers RE, ver. 6.0.2.3, (Thermofisher Scientific, Waltham, MA, United States). The results were interpreted according to the manufacturer’s recommendation.

### IgG assay against SARS-CoV-2 nucleocapsid (N) protein

The levels of IgG against SARS-CoV-2 N protein was determined using the SARS-CoV-2 IgG assay (Alinity, Abbott).

### Whole genome sequencing and genome data analysis

Randomly selected SARS-CoV-2 RT-PCR positive archived clinical specimens were retrieved for the determination of viral lineage with whole genome sequencing using the Oxford Nanopore MinION device (Oxford Nanopore Technologies) as we described previously^29^ (Supplementary Table S1). Nanopore sequencing was performed following the Nanopore protocol—PCR tiling of COVID-19 (Version: PTC_9096_v109_revH_06Feb2020) according to the manufacturer’s instructions with minor modifications (Oxford Nanopore Technologies). Briefly, extracted RNA was first reverse transcribed to cDNA using SuperScript™ IV reverse transcriptase (ThermoFisher Scientific, Waltham, MA, USA). PCR amplification was then performed using the hCoV-2019/nCoV-2019 Version 3 Amplicon Set (Integrated DNA Technologies, Coralville, IA, USA) with the Q5® Hot Start High-Fidelity 2X Master Mix kit (New England Biolabs, Ipswich, Massachusetts, United States) according to the Nanopore protocol. PCR products were purified using 1 × AMPure XP beads (Beckman Coulter, Brea, CA, USA) and quantified using Qubit dsDNA HS Assay Kit (Thermo Fisher Scientific, Waltham, Massachusetts, United States). The purified DNA was then normalized for end-prep and native barcode ligation reactions according to the PCR tiling of COVID-19 virus protocol with Native Barcoding Expansion 96 (EXP-NBD196, Oxford Nanopore Technologies). Barcoded libraries were then pooled, purified with 0.4 × AMPure XP beads and then quantified using Qubit dsDNA HS Assay Kit. Purified pooled libraries were ligated to sequencing adapters and sequenced with the Oxford Nanopore MinION device using R9.4.1 flow cells for 24–48 h.

For bioinformatics analysis, the recommended ARTIC bioinformatics workflow (version 1.2.1) was used with minor modifications applied as described previously^29^. The modifications include reducing the minimum length at the guppyplex step to 350 to allow potential small deletions to be detected and increasing the “–normalise” value to 999,999 to incorporate all the sequenced reads and the high accurate mode was used for basecalling with an increased QC passing score from 7 to 10. The sequence NC_045512.2 obtained from NCBI was used as the reference and the alignment files produced by Medaka were inspected using Integrative Genomics Viewer (IGV) (2.8.0) to verify the mutations called by the ARTIC pipeline. SARS-CoV-2 lineage was assigned using the the online Nextclade tool (Version 2023-03-28)^30^. All sequences were deposited onto the GISAID database (Supplementary Table S2).

### Statistical analysis

Statistical analysis was performed using PRISM 9.4.0 or SPSS 26.0.0. Categorical and continuous variables were compared using Fisher’s exact test and Mann Whitney U test, respectively. One way ANOVA with Dunn’s multiple comparisons test was used for the comparison of > 2 groups. Subgroup analysis was performed to exclude the effect of potential confounding effect of prior infection. The sample size was based on feasibility. Multivariable regression analysis was performed to control for confounding factors, and included KTRs, use of immunosuppressnts, number of immunosuppressants, and use of anti-metabolite immunosuppressant. A *P* value of less than 0.05 was judged as statistically significant.

## Results

### Patient characteristics

Between August 11 and October 5, 2022, we have recruited a total of 89 patients, but one patient was excluded from analysis because of failed T cell assay. The 88 patients in the final analysis included 19 KTRs (Table 1). Overall, the median age was 58 years old (interquartile range [IQR]: 51–71 years old), and the male-to-female ratio was 1:1. GN (44.3% [39/88]) and diabetes mellitus (27.3% [24/88]) were the most common causes of the CKD. Prednisolone was the most frequently used immunosuppressant (40.9% [36/88]), followed by calcineurin inhibitors (30.7% [27/88]) and anti-metabolites (20.5%; 18/88]) (Table 1 and Supplementary Table S3). At the time of patient recruitment, 33% [29/88] had prior SARS-CoV-2 infection, including 25 who had infection between February and June 2022 when Omicron BA.2 sublineage dominated, 2 who had infection between August and September 2022 when Omicron BA.5 sublineage dominated, and 2 who did not report infection but tested positive antibody against the SARS-CoV-2 N protein. Regarding vaccination history, 85.2% (75/88) has completed the primary series (received at least 2 doses) of COVID-19 vaccine, and 95.5% (84/88) either had prior infection or had completed the primary vaccine series.

**Table 1:** Baseline characteristics of study participants.

	All patients (n = 88)	KTRs			Prior infection		
		No^a^ (n = 69)	Yes^b^ (n = 19)	P value^c^	No (n = 59)	Yes (n = 29)	P value^c^
Demographics							
Median age in years (IQR)	58 (51–71)	62 (52–72)	53 (51–58)	0.016	58 (51–70)	63 (51–72)	0.365
Sex							
Female	44 (50)	37 (53.6)	7 (36.8)	0.300	29 (49.2)	15 (51.7)	1.000
Male	44 (50)	32 (46.4)	12 (63.2)		30 (50.8)	14 (48.3)	
Cause of renal failure							
Diabetes mellitus	24 (27.3)	20 (29.0)	4 (21.1)	0.573	14 (23.7)	10 (34.5)	0.316
Hypertension	1 (1.1)	0 (0)	1 (5.3)	0.216	1 (1.7)	0 (0)	1.000
Glomerulonephritis	39 (44.3)	29 (42.0)	10 (52.6)	0.444	29 (49.2)	10 (34.5)	0.255
Others^d^	24 (27.3)	20 (29.0)	4 (21.1)	0.573	15 (25.4)	9 (31.0)	0.616
Immunosuppressant							
Any	39 (44.3)	21 (30.4)	18 (94.7)	< 0.001	25 (42.4)	14 (48.3)	0.652
Prednisolone	36 (40.9)	20 (29.0)	16 (84.2)	< 0.001	23 (39.0)	13 (44.8)	0.649
Calcineurin inhibitors	27 (30.7)	10 (14.5)	17 (89.5)	< 0.001	14 (23.7)	13 (44.8)	0.052
Anti-metabolite	18 (20.5)	6 (8.7)	12 (63.2)	< 0.001	15 (25.4)	3 (10.3)	0.159
mTOR inhibitor	3 (3.4)	0 (0)	3 (15.8)	0.009	2 (3.4)	1 (3.4)	1.000
Single Immunosuppressive drug	7 (8)	7 (10.1)	0 (0)	0.338	6 (10.2)	1 (3.4)	0.418
Combination immunosuppressive drugs	32 (36.4)	14 (20.3)	18 (94.7)	< 0.001	19 (32.2)	13 (44.8)	0.346
KTRs	19 (21.6)	NA	NA	NA	12 (20.3)	7 (24.1)	0.784
Blood test							
Median creatinine (IQR)	694 (131–936)	742 (362–965)	119 (108–158)	< 0.001	656 (135–937)	694 (125–938)	0.918
Median eGFR (ml/min)^e^ (IQR)	8.5 (4.0–48.5)	5.5 (4–25)	53 (38–64)	< 0.001	8 (4–45)	11 (4–48)	0.943
COVID-19 infection							
Infected prior to blood specimen collection	29 (33.0)	22 (31.9)	7 (36.8)	0.784	NA	NA	NA
Vaccine doses							
Received at least 1 dose of COVID-19 vaccine	80 (90.9)	61 (88.4)	19 (100)	0.193	55 (93.2)	25 (86.2)	0.431
Completed primary series	75 (85.2)	56 (81.2)	19 (100)	0.062	55 (93.2)	20 (69.0)	0.008
Received at least 1 booster	53 (60.2)	35 (50.7)	18 (94.7)	< 0.001	41 (69.5)	12 (41.4)	0.020
Vaccine type^e^							
CoronaVac only	30 (37.5)^f^	25 (41.0)^f^	5 (26.3)^f^	0.337	20 (36.4)^f^	10 (40.0)^f^	0.597
BNT162b2 only	43 (53.8)^f^	30 (49.2)^f^	13 (68.4)^f^		29 (52.7)^f,g^	14 (56.0)^f,g^	
BNT162b2 plus CoronaVac	7 (8.8)^f^	6 (9.8)^f^	1 (5.3)^f^		6 (10.9)^f,g^	1 (4.0)^f,g^	
Median number of days between the last dose of vaccination or last episode of infection and specimen collection^h^	130 (84–173)	138 (84–171)	110 (62–196)	0.987	129 (73–171)	131 (90–174)	0.974

Data are n (%) unless otherwise specified.

Abbreviations: eGFR, estimated glomerular filtration rate; IQR, interquartile range; KTRs, kidney transplant recipients.

^a^54 patients are dialysis dependent (29 patients with hemodialysis; 25 patients with peritoneal dialysis).

^b^The median time interval since the last transplantation was 18 years (IQR: 8–26 years).

^c^The P values were calculated using Mann Whitney U test for continuous variables and Fisher’s exact test for categorical variables.

^d^Include polycystic kidney disease, renal angiolipomyoma, obstructive uropathy, CKD of unknown cause.

^e^For the purpose of statistical analysis, a value of 91 was used if the eGFR is greater than 90.

^f^Only included the 80 patients who have received at least one dose of vaccine.

^g^Of the 37 patients who were vaccinated with BTN162b2 but did not report infection, 2 tested positive for antibody against SARS-CoV-2 N protein and were classified as infected.

^h^Excluded 4 patients who were not infected and not vaccinated. For patients in which the date of infection cannot be ascertained, the date of last vaccination was used for the calculation.

### Overall humoral and cellular immune response

First, we determined the genomic epidemiology of SARS-CoV-2 lineages that were circulating at the time of the study in Hong Kong (Supplementary Figure S1). A total of 1572 SARS-CoV-2 specimens were sequenced between January 2022 and February 2023 (Supplementary Table S1). Between January and August 2022, BA.2.2 was the dominant SARS-CoV-2 Omicron sublineage. In September 2022, BA.5.2 became the dominant sublineage. In December 2022 and January 2023, both BA.5.2 and BA.2.3.20 sublineages were prevalent. Therefore, we have chosen BA.2.2, BA.2.3.20 and BA.5.2 for subsequent nAb assays. Furthermore, we have included XBB.1 because the proportion of XBB.1 and its descendants started to increase in February 2023, and XBB.1 were previously found to be much more immunoevasive than BA.5.2^26^.

**Figure 1:**
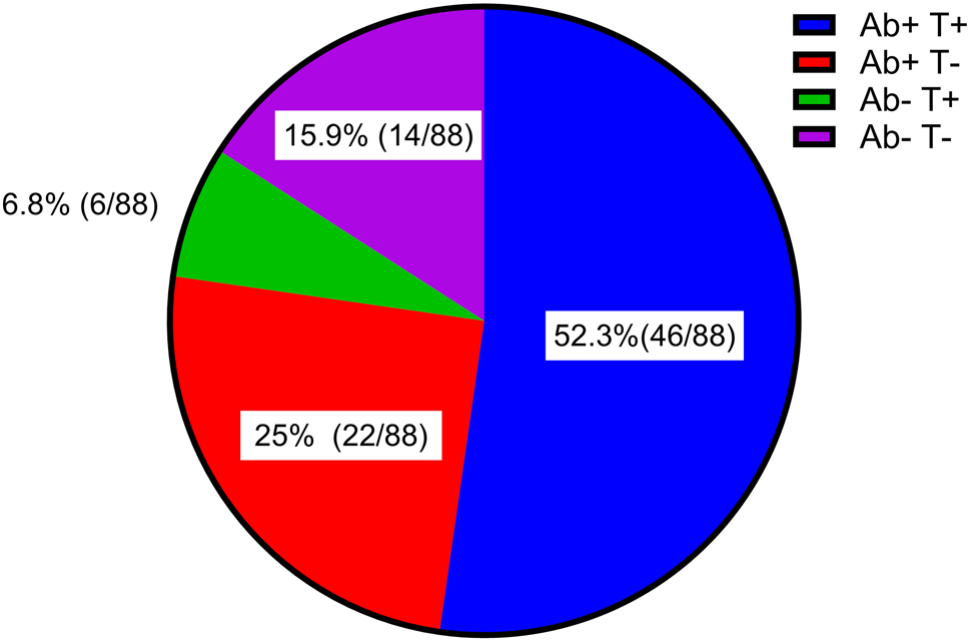
Humoral and cellular immune response among the 88 CKD patients in this study.

Next, we determined the variant-specific nAb titer using a live virus neutralization assay and the SARS-CoV-2-specific T cell response using the IGRA (Fig. 1). 77.3% (68/88) had detectable nAb titer against at least one of the SARS-CoV-2 strains; 59.1% (52/88) tested positive in the IGRA; and 52.3% (46/88) had both detectable nAb and tested positive in the IGRA. However, 15.9% (14/88) did not have detectable levels of nAb against any of the SARS-CoV-2 strains and tested negative in the IGRA.

**Figure 2:**
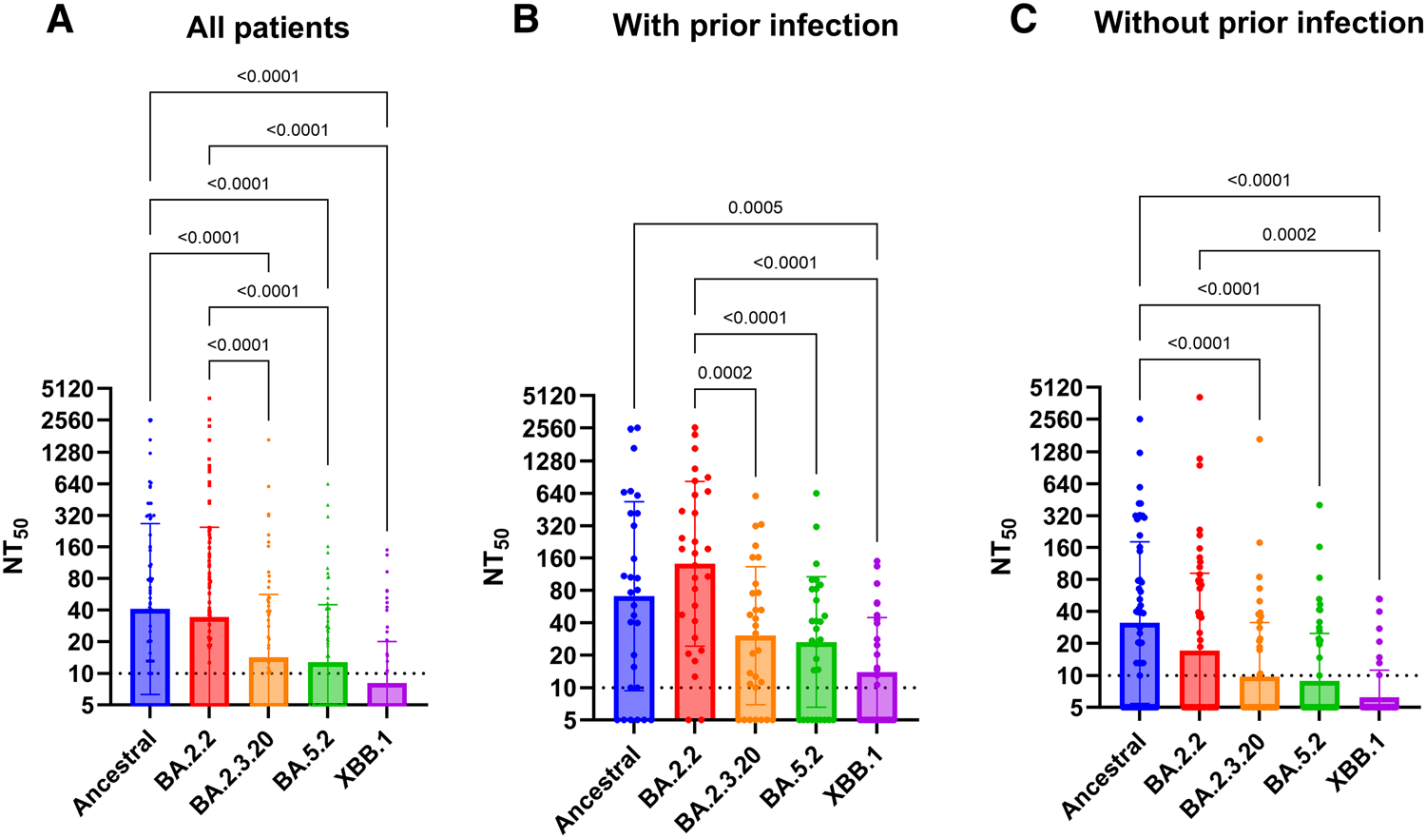
Comparison of live virus neutralizing antibody titers against ancestral strain and Omicron sublineages BA.2.2, BA.2.3.20, BA.5.2 and XBB.1. Dotted horizontal lines represent the lower limit of detection. P values were shown if < 0.05. NT_50_, 50% neutralization titer.

The nAb detection rate was highest for the ancestral strain (70.5% [62/88]), followed by BA.2.2 (59.1% [52/88]), BA.2.3.20 (46.6% [41/88]), BA.5.2 (42% [37/88]), and XBB.1 (26.1% [23/88]) (Table 2). The nAb GMTs against XBB.1 (8.093), BA.5.2 (12.75) or BA.2.3.20 (14.15) were significantly lower than those against the ancestral strain (40.94) and BA.2 (34.29) (*P* < 0.001 for all comparisons) (Fig. 2).

**Table 2:** SARS-CoV-2 neutralizing antibody and T cell response.

Assay	All patients (n = 88)	KTRs			Prior infection		
		No (n = 69)	Yes (n = 19)	*P* value	No (n = 59)	Yes (n = 29)	*P* value^a^
NAb detected							
Ancestral	62 (70.5)	51 (73.9)	11 (57.9)	0.255	39 (66.1)	23 (79.3)	0.226
BA.2.2	52 (59.1)	42 (60.9)	10 (52.6)	0.602	25 (42.4)	27 (93.1)	< 0.001
BA.2.3.20	41 (46.6)	32 (46.4)	9 (47.4)	1.000	19 (32.2)	22 (75.9)	< 0.001
BA.5.2	37 (42)	28 (40.6)	9 (47.4)	0.610	17 (28.8)	20 (69.0)	0.001
XBB.1	23 (26.1)	18 (26.1)	5 (26.3)	1.000	8 (13.6)	15 (51.7)	< 0.001
IGRA positive	52 (59.1)	42 (60.9)	10 (52.6)	0.602	31 (52.5)	21 (72.4)	0.106
nAb not detected^b^ & IGRA negative	14 (15.9)	7 (10.1)	7 (36.8)	0.010	13 (22.0)	1 (3.4)	0.030

Data are n (%).

Abbreviations: IGRA, interferon gamma release assay; nAb, neutralizing antibody.

^a^Fisher’s exact test.

^b^Neutralizing antibody not detected against ancestral strain, BA.2.2, BA.2.3.20, BA.5.2 and XBB.1.

### Difference in nAb and T cell response between individuals with or without prior infection

**Figure 3:**
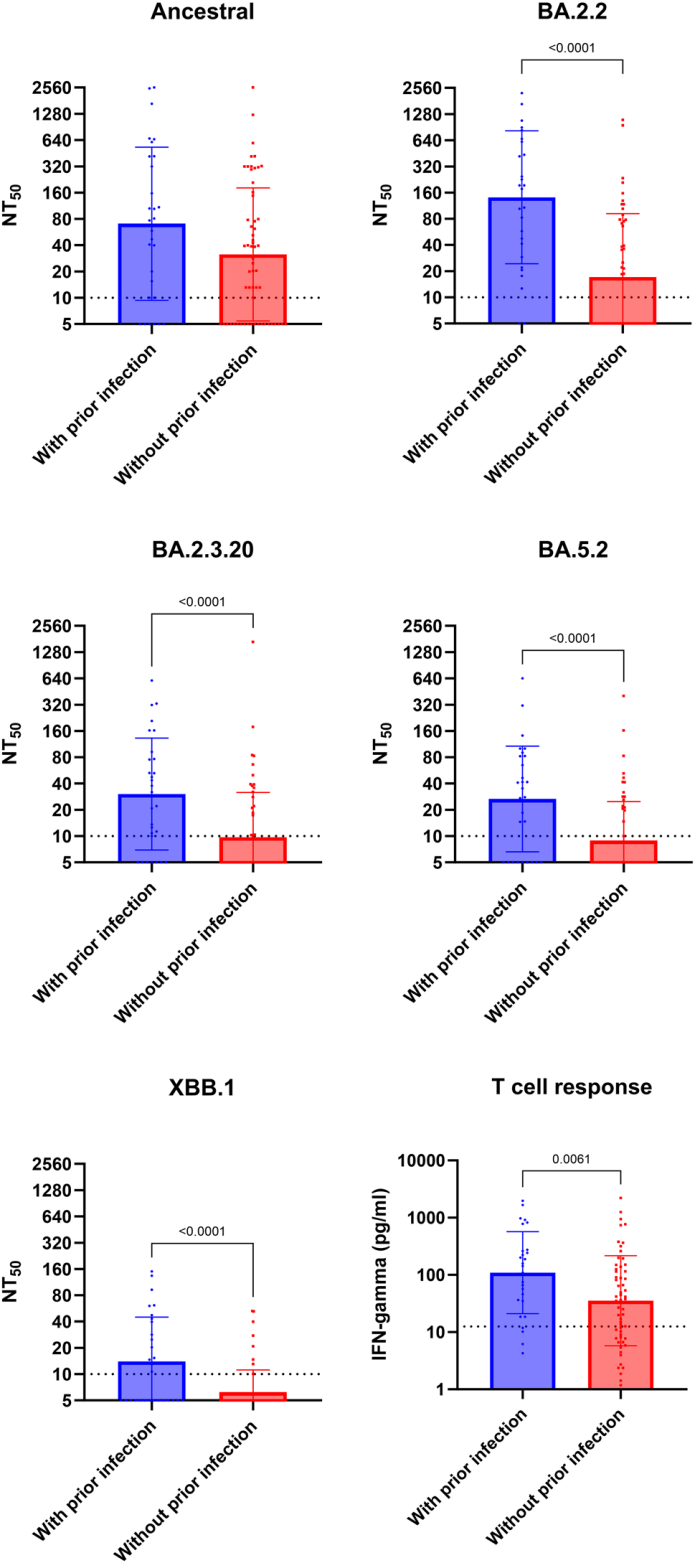
Comparison of live virus neutralizing antibody titers and T cell response between patients who had prior infection and those without prior infection. T cell response was determined by interferon gamma release assay. Dotted horizontal lines represent the lower limit of detection. *P* values were shown if < 0.05. NT_50_, 50% neutralization titer.

Previous studies suggested that there was a significant difference in nAb and T cell response between previously infected and non-infected patients^11^. Therefore, we analyzed the effect of prior infection on nAb and T cell response. The baseline characteristics of patients with or without prior infection were similar, except that those without prior infection had a higher proportion who have completed primary series (93.2% [55/59] versus 69.0% [20/29]; *P* = 0.008) and received at least one vaccine booster dose (69.5% [41/59] versus 41.4% [12/29]; *P* = 0.020) than those with prior infection (Table 1). Individuals with prior infection had significantly higher rate of nAb detection for BA.2.2 (93.1% [27/29] versus 42.4% [25/59]; *P* < 0.001), BA.2.3.20 (75.9% [22/29] versus 32.2% [19/59]; *P* < 0.001), BA.5.2 (69.0% [20/29] versus 28.8% [17/59]; *P* < 0.001), and XBB.1 (51.7% [15/29] versus 13.6% [8/59]; *P* < 0.001) than those without prior infection (Table 2). However, there was no significant difference in the nAb detection rate for ancestral strain between patients with and those without prior infection (79.3% [23/29] versus 66.1% [39/59]; *P* = 0.226). Individuals with prior infection also had a significantly higher nAb titers against all Omicron sublineage viruses (BA.2.2 [*P* < 0.0001], BA.2.3.20 [*P* < 0.0001], BA.5.2 [*P* < 0.0001], XBB.1 [*P* < 0.0001]) than non-infected individuals, but there was no statistically significant difference for ancestral strain (Fig. 3). The IFN gamma response was also more robust among participants with prior infection than those without prior infection (*P* = 0.0061) (Fig. 3). The proportion of patients without any nAb or T cell response against SARS-CoV-2 were higher among patients without prior infection than those with prior infection (22.0% [13/59] versus 3.4% [1/29]; *P* = 0.030) (Table 2 and Supplementary Figure S2).

### Difference in nAb and T cell response between KTR and non-KTR CKD patients

Previous studies showed that KTRs have poorer immune response against SARS-CoV-2. Therefore, we performed a subgroup analysis to determine if there were any differences in the nAb and T cell response between KTRs and non-KTRs. For the comparison between baseline characteristics of the groups, the KTR group was significantly younger than the non-KTR group (median age: 53 [IQR, 51–58] versus 62 [IQR, 52–72]; *P* = 0.016) (Table 1). A significantly higher proportion of KTRs require immunosuppressant than non-KTRs (*P* < 0.001). Furthermore, a significantly higher proportion of KTRs receive vaccine booster doses than non-KTRs (*P* < 0.001).

There was no significant difference in the nAb titers or IFN-gamma response between the KTR and non-KTR groups (Supplementary Figure S3, and Table 2), and between KTR and the non-KTR who are not taking immunosuppressive drugs (Supplementary Figure S4). However, 36.8% of KTRs did not have detectable levels of nAb and tested negative in the IGRA, which were significantly higher than that of non-KTRs (10.1%) (Table 2).

### Difference in nAb and T cell response between patients on immunosuppressant and those not on immunosuppressants among CKD patients without prior infection

Immunosuppressants can suppress nAb and T cell response. To determine the effect of immunosuppressant among CKD patients, we compared the nAb and T cell response between patients taking immunosuppressants and those not taking immunosuppressants. To avoid the effect of prior infection, we limited our analysis to 59 patients without prior infection. The geometric mean nAb titers against BA.2.2 (*P* = 0.0441) and XBB.1 (*P* = 0.0160) were significantly lower among patients taking immunosuppressants than those not taking immunosuppressants (Supplementary Figure S5), but there was no statistically significant difference in the T cell response.

Since most KTR patients are taking immunosuppressants, we have performed a multivariable regression analysis to determine independent factors associated with nAb response. We included the following variables: KTR, use of immunosuppressant, number of immunosuppressants, and use of anti-metabolite immunosuppressant. Only KTR remained to be an independent risk factor for the absence of nAb response against any of SARS-CoV-2 strains tested (*P* = 0.007).

### Correlation of protection

**Figure 4:**
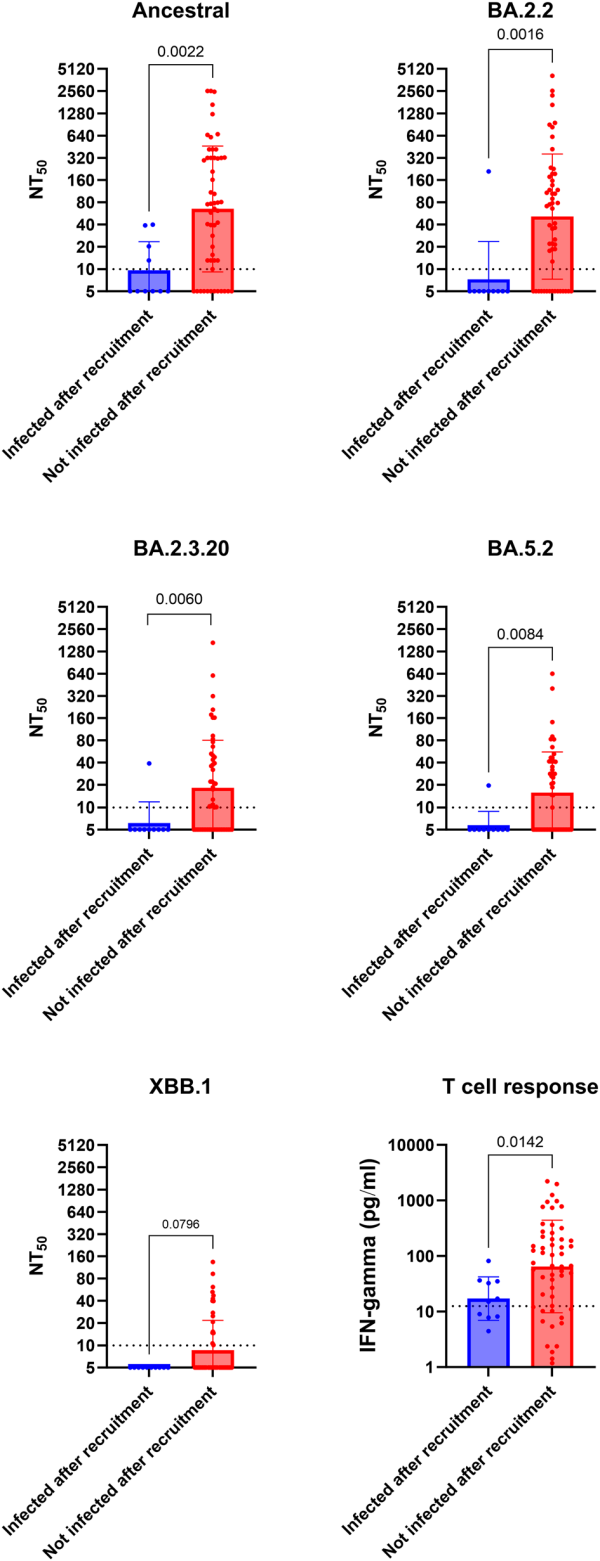
Comparison of live virus neutralizing antibody titers and T cell response between patients who had subsequent infection and those without subsequent infection after recruitment. T cell response was determined by interferon gamma release assay. Dotted horizontal lines represent the lower limit of detection. *P* values were shown if < 0.05. NT_50_, 50% neutralization titer.

Next, we determined whether the nAb titer or IFN-gamma response correlate with protection. For this analysis, we excluded 19 individuals who received COVID-19 vaccine after recruitment and 5 individuals who died within 120 days after recruitment. Out of the remaining 64 individuals, 10 (15.6%) were infected within 120 days after recruitment. The levels of nAb (against ancestral strain, BA.2.2, BA.5.2, BA.2.3.20) and T cell response were significantly lower in patients who had infection within 120 days of recruitment (Fig. 4). Since prior infection was almost statistically associated with protection (*P* = 0.050; Table 3), we specifically determined whether the levels of nAb and T cell response were associated with protection among individuals who did not have prior infection. We found that nAb against ancestral strain and BA.2.2 remain statistically significant (Supplementary Figure S6).

**Table 3:** Factors associated with infection within 120 days of after recruitment.

	Non-infected (n = 54)	Infected (n = 10)	*P* value^a^
Median age in years (IQR)	57 (49–70)	56 (50–67)	0.890
Sex			
Female	28 (51.9)	3 (30)	0.305
Male	26 (48.1)	7 (70)	
KTR	15 (27.8)	3 (30)	1.000
Receive immunosuppressant	26 (48.1)	5 (50)	1.000
Received vaccine booster	41 (75.9)	8 (80)	1.000
Had prior infection	18 (33.3)	0 (0)	0.050

Data represent no. (%) unless otherwise specified.

Abbreviations: GMT, geometric mean titer; IFN, interferon; IQR, interquartile range; KTR, kidney transplant recipient; nAb, neutralizing antibody.

^a^Fisher’s exact test.

## Discussion

Previous studies suggest that both humoral and cellular immunity are important for protection against SARS-CoV-2 infection^7–9^. Patients with CKD, especially those receiving immunosuppressive treatments, have various defects in both arms of immunity, and hence show increased risk of severe COVID-19 infections^13^. Here, we first conducted a cross-sectional study to assess the humoral and T cell immunity among a cohort of CKD patients in August-October 2022, following the BA.2.2 wave in early 2022 and amidst the BA.5.2 dominant wave. We found that despite 95% of study participants have been exposed to either natural SARS-CoV-2 infection or received a full course of COVID-19 vaccination, 15.9% of patients did not have detectable nAb or T cell response against SARS-CoV-2. It was particularly worrisome for KTRs, as 36.8% showed no detectable nAb or T cell response, despite all of them had either prior infection or received booster doses of COVID-19 vaccines.

It is well recognized that proper cognate B cell-T cell interaction, followed by their differentiation and maturation, are prerequisite for robust immune response and immunological memory. KTRs generally receive triple immunosuppression comprising corticosteroids, CNI and anti-metabolites (e.g. MMF), thereby inhibiting multiple critical steps in B and T cell activation/differentiation, and thus exhibit significantly poorer rates of detectable nAb or T cell response than non-KTRs. Indeed, these observations are in line with the results of our recent meta-analysis, which showed that KTRs had significantly lower seropositive rates after COVID-19 vaccination than non-KTR patients, and the lower response rates might be related to the use of MMF that had a negative influence on the development of anti-spike antibodies^14^.

XBB is one of the most immunoevasive Omicron sublineage^26^. Data from our cohort of CKD patients showed that nAb titer against XBB.1 was the lowest among all variants in individuals with or without prior infection. Since XBB has become the dominant sublineage worldwide since April 2023^31^, we envisage that CKD patients, especially KTRs, will be highly susceptible to the currently circulating strain and potentially its serious complications.

In this study, we separately analyzed the nAb against different SARS-CoV-2 variants and T cells response in patients with or without prior infections as hybrid immunity can affect both B cell and T cell responses^11,32^. Whilst nAb titers against the ancestral strain were similar between patients with or without previous infection, the former group showed significantly higher nAb against the other strains including BA.2.2, BA.2.3.20, BA.5.2 and XBB.1. Moreover, patients with previous infection also showed better T cell response than those without prior infections. These observations highlight the significance of hybrid immunity in CKD patients who have impairment in both B and T cell responses.

We found that nAb titers and T cell response were significantly associated with infection within 120 days after recruitment. Prior infection also appeared to show protection within 120 days after recruitment, almost reaching statistical significance (*P* = 0.050). These results concur with those of previous studies^33^, and demonstrate the importance of including nAb and T cell response in predicting the risk of infection, especially for patients without prior infection. In contrast, patient characteristics alone such as transplant status, use of immunosuppressants or reception of booster vaccine did not appear to be adequate for predicting the risk of infection within 120 days after recruitment in CKD patients. While nAb and T cell response assays are labor-intensive and costly, it may be worthwhile to evaluate these important immunological functions in CKD patients, especially KTRs, as more than one third of them would show absent response which are predictive of the risk for subsequent COVID-19 infections. Furthermore, future studies are required to establish the protective threshold of nAb and T cell response among different subgroups of CKD patients.

There are several limitations in this study. First, within each of the cohorts, there is heterogeneity in the patients’ characteristics. Our sample size is not sufficiently powered to delineate the impact of each of these confounding factors on the different outcome measures. For instance, we have previously performed our analysis with sub-categorization of the non-KTR group into patients on PD, patients on HD and those not requiring dialysis. However, due to the small number in each group after this subdivision, the analysis was underpower to draw meaningful conclusions. Along the same line, our study was also under-powered to examine the impact of different combinations of immunosuppressive drugs on nAb and T cell response. Second, although our patients had frequent COVID-19 testing as part of the mandatory requirement of the hospital administration for patients to attend dialysis sessions and out-patient follow-up visits, we cannot completely rule out the possibility of subclinical infection for patients classified as not having prior infection. Third, we cannot rely on anti-nucleoprotein antibody as a biomarker of prior infection as many patients received the CoronaVac, an inactivated whole virion vaccine. Fourth, the IGRA that we used in this study cannot differentiate CD4 + and CD8 + T cell response.

Our findings have important clinical implications as a considerable proportion of KTRs remain highly vulnerable to circulating strains/variants of SARS-CoV-2 despite previous infection or a full course of vaccination. Better vaccination strategies are required. For example, we may need to consider studying the intradermal route of vaccination and the use of adjuvants. Our previous study demonstrated that pretreatment with topical imiquimod before intradermal hepatitis B vaccine could significantly enhanced the seroprotection rate among CKD patients on dialysis^34^. Another strategy is intranasal vaccination, which can improve the mucosal immunity^35,36^. Furthermore, we need to consider the possibility of post-exposure prophylaxis among these at-risk individuals.

## Author contributors

D.Y.H.Y., C.H.Y.F., X.Z. and K.K.W.T. contributed to the conception and design of the study. D.Y.H.Y., C.H.Y.F., X.Z., J.D.I., W.M.C., A.W.H.C., L.L.C., Y.Z., B.P.C.C. contributed to data acquisition. D.Y.H.Y., C.H.Y.F., X.Z., J.D.I., and K.K.W.T. contributed to data analysis. D.Y.H.Y., C.H.Y.F., X.Z., J.D.I., T.M.C., K.S.L., V.C.C. and K.K.W.T. contributed to data interpretation. K.K.W.T. had roles in funding acquisition. D.Y.H.Y. and K.K.W.T. had verified the underlying data. All authors reviewed and edited the manuscript. D.Y.H.Y., C.H.Y.F., X.Z. and K.K.W.T. had full access to the data in the study, and have shared the responsibility for the decision to submit for publication.

### Supplementary Information

Supplementary Figures.

Supplementary Table S1.

Supplementary Table S2.

Supplementary Table S3.

## Data Availability

The datasets used and/or analysed during the current study available from the corresponding author on reasonable request.
