# Supplementary material for: Humoral and cellular immunity against different SARS-CoV-2 variants in patients with chronic kidney disease
Source: Sci Rep. 2023 Nov 15;13:19932. doi: 10.1038/s41598-023-47130-8 (PMC10652016; doi:10.1038/s41598-023-47130-8)
Supplement: Supplementary file 1 — Supplementary Figures. [file 41598_2023_47130_MOESM1_ESM.docx]

**SUPPLEMENTARY FIGURES**


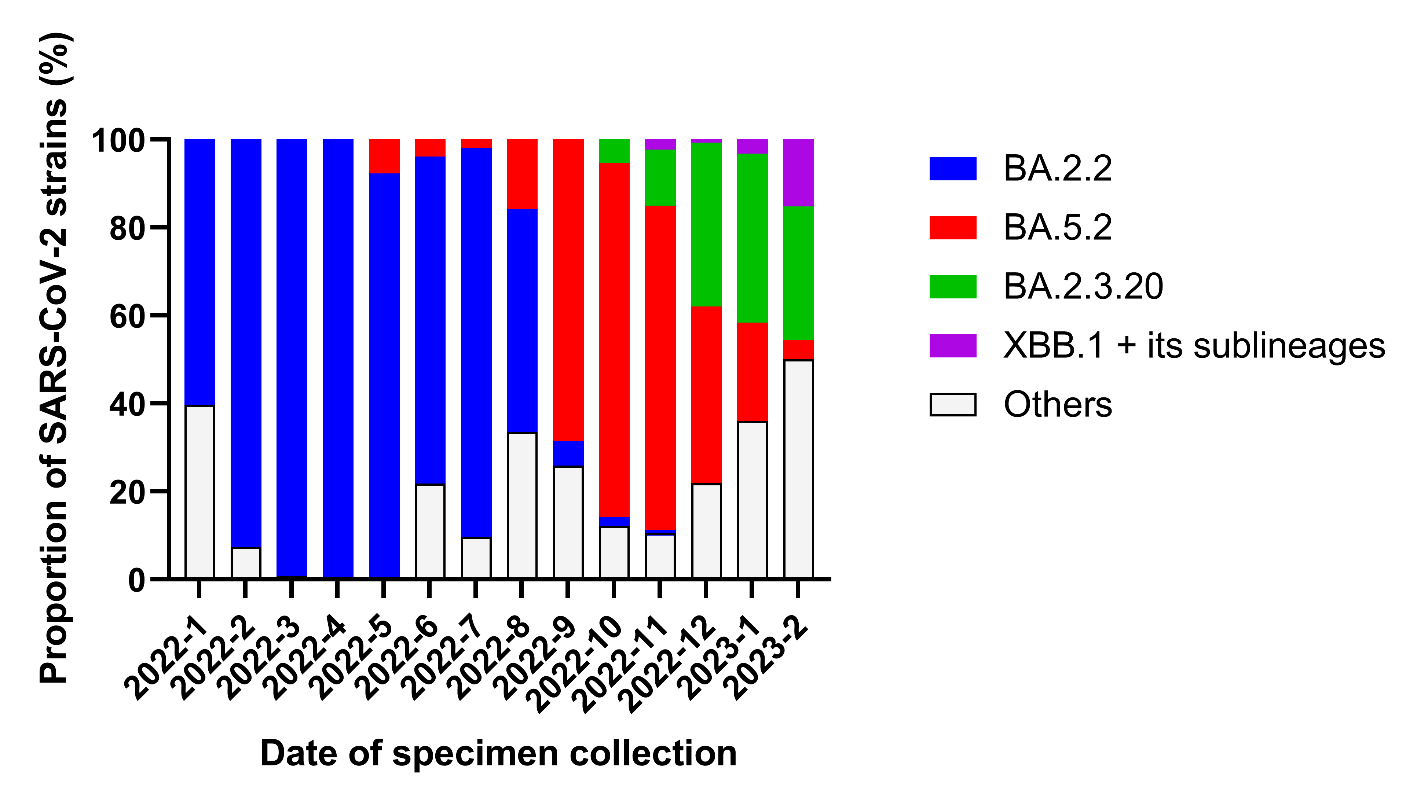
 **Supplementary Figure S1.** Proportion of SARS-CoV-2 variants during the study period.

**Supplementary Figure S2.** Humoral and cellular immune response among the 88 CKD patients in this study.


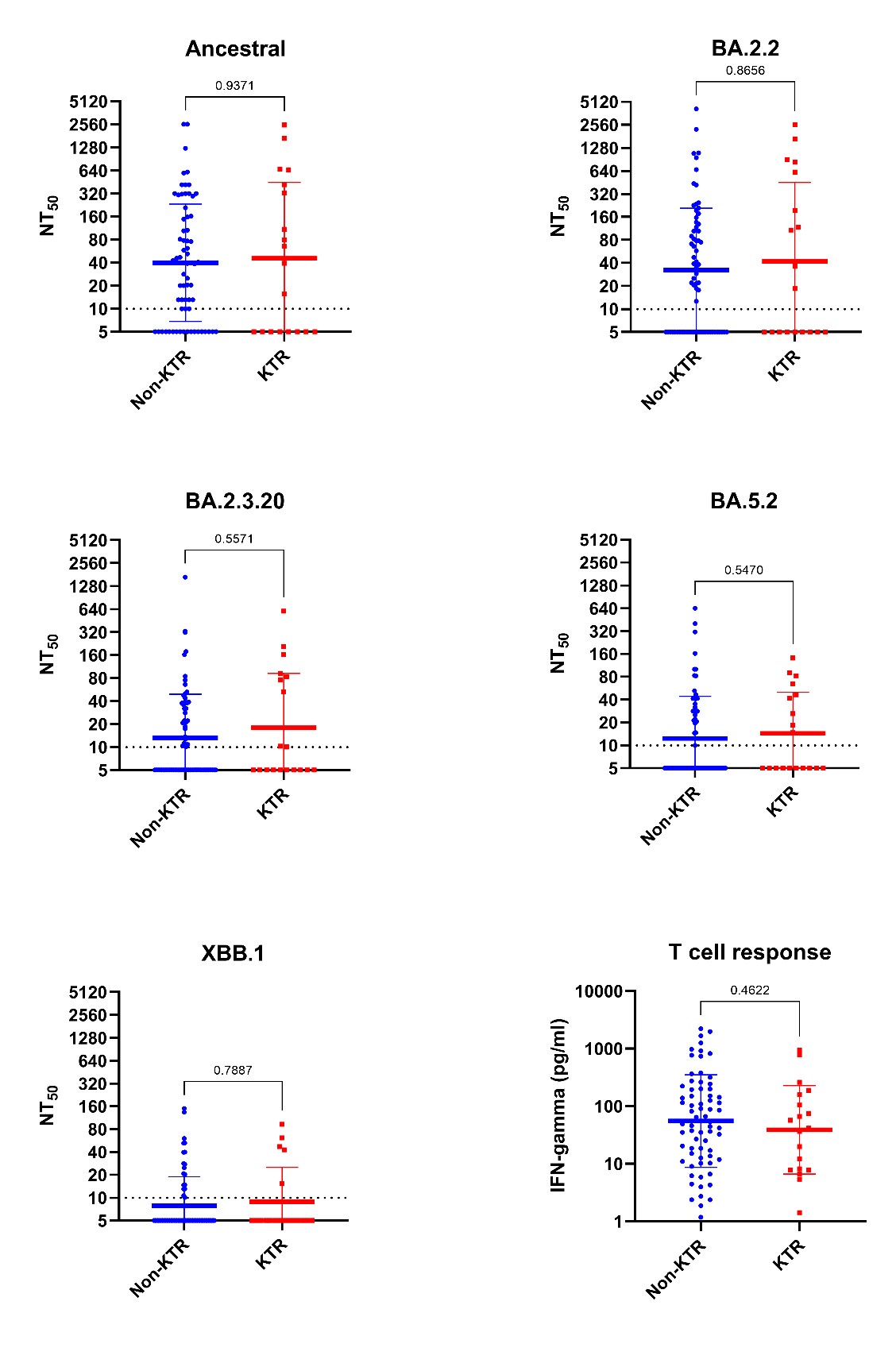


**Supplementary Figure S3**. Comparison of live virus neutralizing antibody titers and T cell response between kidney transplant recipients (KTRs) and non-KTRs. T cell response was determined by interferon gamma release assay. Mann Whitney test was used for statistical analysis. Dotted horizontal lines represent the lower limit of detection. P values were shown if <0.05. NT_50_, 50% neutralisation titer.

**Supplementary Figure S4**. Comparison of live virus neutralizing antibody titers and T cell response between kidney transplant recipients (KTRs) and non-KTRs who are not taking immunosuppressive drugs. T cell response was determined by interferon gamma release assay. Mann Whitney test was used for statistical analysis. Dotted horizontal lines represent the lower limit of detection. P values were shown if <0.05. NT_50_, 50% neutralisation titer.

**Supplementary Figure S5**. Comparison of live virus neutralizing antibody titers and T cell response between patients taking immunosuppressants and those not taking immunosuppressants. T cell response was determined by interferon gamma release assay. Mann Whitney test was used for statistical analysis. Dotted horizontal lines represent the lower limit of detection. P values were shown if <0.05. IS, immunosuppressant; NT_50_, 50% neutralisation titer.


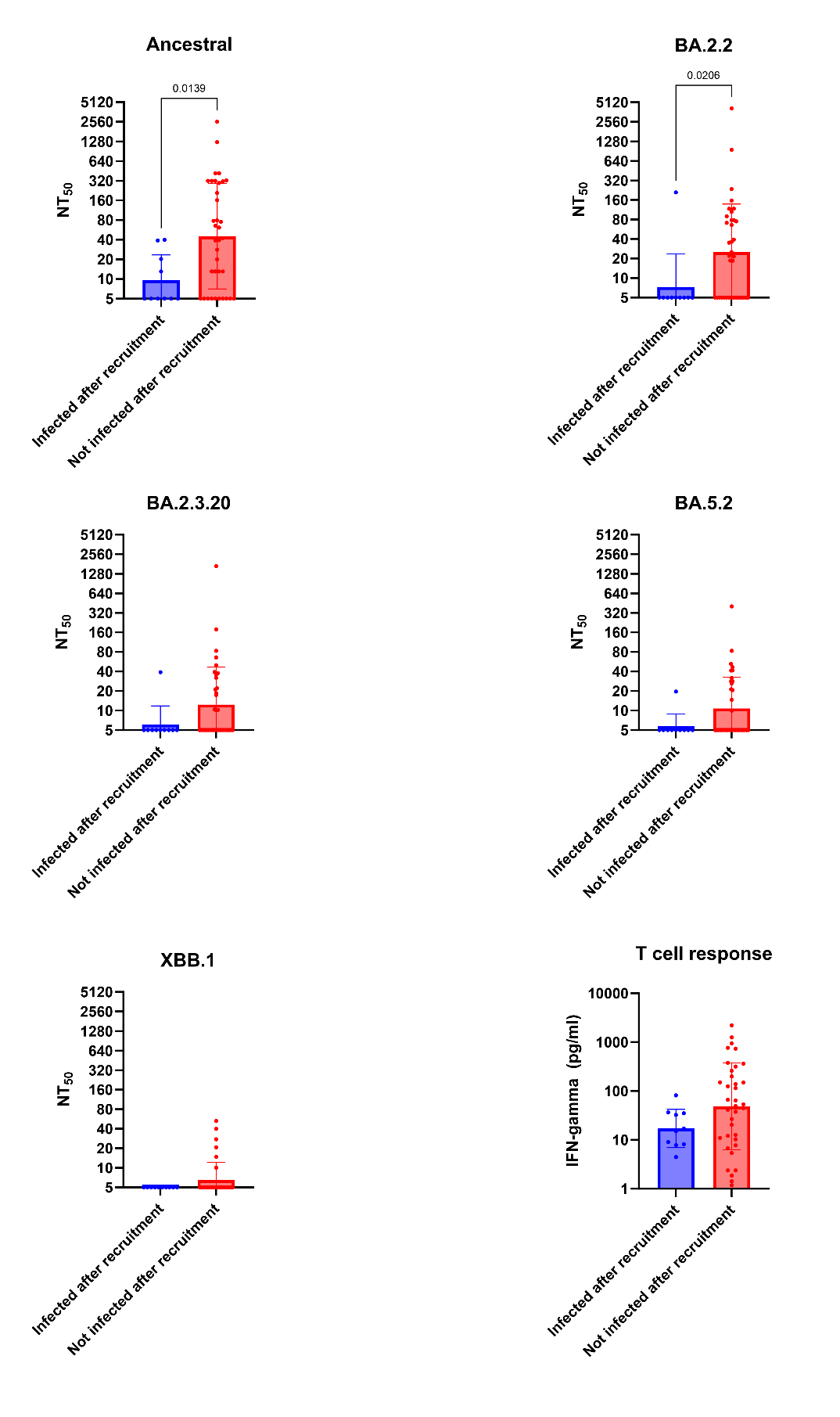


**Supplementary Figure S6**. Comparison of live virus neutralizing antibody titers and T cell response between patients who had subsequent infection and those without subsequent infection after recruitment. Only patients without infection before recruitment were included in this figure. T cell response was determined by interferon gamma release assay. Mann Whitney test was used for statistical analysis. Dotted horizontal lines represent the lower limit of detection. P values were shown if <0.05. NT_50_, 50% neutralization titer.
