# Supplementary material for: Humoral and cellular immunity against different SARS-CoV-2 variants in patients with chronic kidney disease
Source: Sci Rep. 2023 Nov 15;13:19932. doi: 10.1038/s41598-023-47130-8 (PMC10652016; doi:10.1038/s41598-023-47130-8)
Supplement: Supplementary file 2 — Supplementary Table S1. [file 41598_2023_47130_MOESM2_ESM.docx]

**Supplementary Table S1.** Number of SARS-CoV-2 sequences included in Figure 1.

| **Year-Month** | **Number of sequences** |
| --- | --- |
| 2022-1 | 202 |
| 2022-2 | 189 |
| 2022-3 | 120 |
| 2022-4 | 36 |
| 2022-5 | 13 |
| 2022-6 | 78 |
| 2022-7 | 104 |
| 2022-8 | 152 |
| 2022-9 | 89 |
| 2022-10 | 148 |
| 2022-11 | 133 |
| 2022-12 | 137 |
| 2023-1 | 125 |
| 2023-2 | 46 |
| Total | 1572 |
